# Isinglass Polysaccharides Regulate Intestinal-Barrier Function and Alleviate Obesity in High-Fat Diet Mice through the HO-1/Nrf2 Pathway and Intestinal Microbiome Environment

**DOI:** 10.3390/nu14193928

**Published:** 2022-09-22

**Authors:** Guopeng Li, Shugang Li, Huanhuan Liu, Lihua Zhang, Jingzhu Gao, Siteng Zhang, Yue Zou, Xiaodong Xia, Xiaomeng Ren

**Affiliations:** Collaborative Innovation Center of Seafood Deep Processing, National Engineering Research Center of Seafood, School of Food Science and Technology, Dalian Polytechnic University, Dalian 116304, China

**Keywords:** crude isinglass polysaccharides (CIP), intestinal barrier, inflammation, HO-1/Nrf2 pathway, intestinal flora

## Abstract

Plant polysaccharide intervention has shown significant potential to combat obesity. However, studies on animal polysaccharides are indeed rare. The aim of this study was to investigate the potential functions of CIP (IL) on obesity, intestinal microflora dysbiosis, and the possible protection of intestinal barrier in mice fed with high-fat diet (HFD). Our results revealed that after 13 weeks, the HFD+L (high-fat diet + 25 mg/kg CIP) group showed significantly more weight loss and fat accumulation relative to the HFD+H (high-fat diet + 50 mg/kg CIP) group. Furthermore, CIP intervention modulated lipid metabolism and mRNA levels of inflammatory mediators in liver. Overall, CIP clearly improved the intestinal barrier in HFD-fed mice. Additionally, we observed that CIP intervention improved intestinal microbiota community richness and diversity in HFD-fed mice. The CIP intervention mice group showed a relatively low *Firmicutes* to *Bacteroidetes* ratio compared to the HFD group. This study concluded that CIP could be used as a functional food to prevent adipocyte accumulation, reduce systemic inflammation, and protect the intestinal barrier.

## 1. Introduction

Obesity has been classified as multifactorial due to the contribution of many risk factors, which include unhealthy eating habits, lack of exercise, and genetics [1]. Overtime, the incidence of non-alcoholic fatty liver disease, type 2 diabetes, coronary heart disease, and ischemic stroke caused by obesity has greatly increased [2]. Obesity has been shown to be a rising threat to human health that has placed tremendous strain on the public health systems [3]. Therefore, controlling and improvement of obesity have been widely a challenge for both individuals and caregivers.

Obesity is strongly associated with the abnormal size and number of white adipocyte cells [4]. Adipose tissue is a central node in the interstitial network formed by adipokines and is a highly dynamic endocrine tissue [5]. Although adipose tissue can induce a variety of favorable effects, including regulation of angiogenesis, metabolism, and inflammation, the excess adipose tissue production, or secretion of adipokines may contribute to obesity-related metabolic diseases [6]. Adipokines are rich in pro-inflammatory mediators, which have been shown to promote disease progression [7]. Therefore, controlling the expression of pro-inflammatory cytokines is important to increase susceptibility to obesity-related inflammation [8].

Chronic systemic inflammation is associated with interactions between the gut barrier and the redox system [9]. Emerging evidence suggests that antioxidants play an important role in preventing systemic chronic inflammation, mainly due to *Nrf2*, which protects the gut barrier by activating tightly bound gut proteins, such as *ZO-1* and *Occludin* [10]. In addition, compounds derived from microorganisms, including food metabolites and short-chain fatty acids (SCFAs), may improve the intestinal barrier [11].

SCFAs, as an end-product of carbohydrate fermentation in the gut microbiome, have been identified to be involved in ameliorating host metabolic syndrome [12]. The role of SCFAs as an additional energy source is contrary to adipogenic factors, demonstrating that SCFAs have some beneficial effects in obesity prevention, including regulating lipid metabolism [13], promoting energy homeostasis [14], anticancer activity [15], and controlling appetite [16]. Intestinal microbiota is an emerging factor affecting obesity and metabolic homeostasis in mammals [17,18]. The human gut contains a variety of microbes that play an important role in the well-being of their hosts [19]. A recent study suggests that dysfunction in the function and structure of the gut microbiome increases the risk of obesity and its complications [20].

From a public health and economic perspective, preventing or treating obesity is of great importance. Dietary therapies are becoming increasingly popular among the general population, where these novel treatments based on diets rich in bioactive foods have attracted the attention of nutritionists and biomedical researchers [21].

Currently, many polysaccharides and their derivatives have been used in various pharmaceutical applications [22]. Most of them are plant-based polysaccharides, which are relatively non-toxic and do not cause serious side effects. However, the effects of animal polysaccharides on HFD-induced obesity and modulation of mice microbiota have rarely been reported. The aim of this study was to evaluate the potential of alum polysaccharides to combat obesity in a mouse model of HFD-induced obesity. In addition, blood lipids, hormones, liver function, intestinal barrier, hepatic lipid, and antioxidants were measured. To elucidate the anti-obesity mechanism, this study systematically analyzed the effects of CIP on the inflammatory response, mice microbiota, and its metabolites, as well as the relationship between different parameters in HFD-induced mice.

To evaluate the efficacy of CIP against obesity, different doses (25 and 50 mg/kg) were administered. At 50 mg/kg, an increase in the release of inflammatory cytokines was observed, which affected intestinal tight junction protein expression. Therefore, we can consider a dose of 25 mg/kg to be optimal.

## 2. Materials and Methods

### 2.1. Preparation of Isinglass Polysaccharides

In the preparation of isinglass polysaccharide, fish isinglass was degreased with acetone. Then, the defatted isinglass was dried and ground into powder. Briefly, 30 g of isinglass was mixed with deionized water at a ratio of 1:30 *w*/*v* and extracted with 4% pepsin at 37 °C and pH = 4.5 for 2 h. After adjusting the pH to 8, 4% of trypsin was added and extraction was performed at 37 °C for 2 h. Finally, the pH was adjusted back to neutral and the enzyme was inactivated at 80 °C for 1 h. Subsequently, the mixture was centrifuged at 8000× *g* for 20 min. After collecting the supernatant and concentrating to a certain volume with a rotary evaporator, 4 times the volume of ethanol was added to the concentrated solution to make the final concentration reach 80% (*v*/*v*). Precipitate formation occurred at 4 °C for 24 h and was collected by centrifugation, then it was dissolved in distilled water. Deproteinization was performed using the Sevag reagent method. Finally, the deproteinized fraction was dialyzed against a 3500 D dialysis bag and lyophilized to yield crude isinglass polysaccharide (CIP).

### 2.2. Basic Physicochemical Properties of CIP

#### 2.2.1. Monosaccharide Analysis and Molecular Weight Identification

High performance liquid chromatography (Waters Co., Milford, MA, USA) was used to estimate the relative molecular mass of CIP [23].

#### 2.2.2. Determination of Antioxidant Activity of CIP

The ABTS, DPPH, and hydroxyl radical scavenging activities of CIP were determined [24].

### 2.3. Animal Study

C57BL/6 male mice (6 weeks old, *n* = 48) were obtained from Changsheng Biotechnology Co., Ltd. (Changchun, China). Mice were maintained in a room at a temperature of 25 °C and relative humidity of 60%. The Animal Ethics Committee of Dalian University of Technology approved all animal experiments, and the experiments were carried out in accordance with the Guidelines of the National Institute for Animal Experiments. After 1 week of acclimation, the experimental subjects were categorized into 4 groups (n = 12) comprised of the Control group, HFD group, HFD+L group, and HFD+H group. Mice were administered intragastrically for 3 weeks after acclimatization. The normal control group (normal) mice were fed the control diet (10% kcal from fat, TP2330055MC, Nantong Trophy Feed Technology Co., LTD., Nantong, China) and gavaged with 200 μL of normal saline. The model group (model group) mice were fed a high-calorie diet (60% kcal from fat, TP230055M, Nantong Trophy Feed Technology Co., LTD.) and gavaged with 200 μL of normal saline. The CIP+L treatment group (HFD-low) mice were fed a high-calorie diet and gavaged with 200 μL of 25 mg/kg CIP. The CIP+H treatment group (HFD-high) mice were fed a high-calorie diet and gavaged with 200 μL of 50 mg/kg CIP. During the experiment, mice were weighed every week and food and fluid intakes were recorded two times per week. After 11 weeks, mice were fasted overnight, anesthetized, and removed retrogradely from the orbital sinus. Then, mice were sacrificed by cervical dislocation. The liver, spleen, groin, and epididymis tissues of mice were weighed and stored at −80 °C.

### 2.4. Histological Analysis

Epididymal adipose, brown adipose, and liver tissues were fixed with 4% neutral paraformaldehyde for 1 day, then paraffin-embedded and sliced at 5 μm. Thereafter, tissue sections were stained with hematoxylin and eosin (H&E) and histopathological features were observed by microscope (Nikon Eclipse TI-S, Tokyo, Japan). Finally, a conventional morphological evaluation was performed.

### 2.5. Biochemical Analysis

Measurements of serum tumor necrosis factor alpha (*TNF-α*) and interleukin 10 (*IL-10*) levels were obtained using commercial ELISA kits (Jiancheng Bioengineering Institute, Nanjing, China). Serum lipopolysaccharide (*LPS*), leptin, adiponectin (*ADP*), total cholesterol (*TCHO*), triacylglycerol (*TG*), high-density lipoprotein cholesterol (*HDL-C*), low-density lipoprotein cholesterol (*LDL-C*), aspartate aminotransferase (*AST*), glutamic-aminotransferase (*ALT*), liver malondialdehyde (*MDA*), protoglutathione (*GSH*), total oxide dismutase (*T-AOC*), and catalase (*CAT*) were determined by a commercial kit (Jiancheng Bioengineering Institute, Nanjing, China).

### 2.6. Quantitative RT-PCR Analysis

Total RNA of liver samples was extracted using Trizol reagent (Shanghai Sangon Biotechnology Co., Ltd., Shanghai, China), and cDNA samples were synthesized with PrimeScript™ RT Kit (cDNA Eraser) (Beijing Bao Biomedical Technology Co., Ltd., Beijing, China). The mRNA expression levels were quantified by the quantitative RT-PCR system with TB Green^®^Premix Ex Taq™II Kit (RR820A, Takara, Beijing, China). The relative gene expression levels of target genes were normalized to reference levels of actin expression and calculated using the 2−ΔΔCt formula.

### 2.7. Western Bolt Analysis

Liver, colon, and adipose tissues were homogenated at 4 °C in RIPA buffer (Thermo Pierce, Waltham, MA, USA) containing protease and phosphatase inhibitors. The homogenate was centrifuged at 12,000× *g* for 10 min at 4 °C and the supernatant (whole tissue extract) was removed for analysis. The protein content of the supernatant was determined by the BCA method (Bio-RAD, CA, USA), and (approximately 60 μg) were separated by SDS-PAGE. Then, it was coated on the PVDF membrane and blocked with 5% (*w*/*v*) skim milk for 1 h at 25 °C. Next, the primary antibody was added to the membrane-containing mixture and incubated overnight at 4 °C. After incubation, the membranes were washed 3 times with TBST for 15 min each and incubated with a secondary antibody (1:3000 dilution) for 1 h. After three TBST washes, bands were visualized with the Bio-Rad Image Analysis System (Berkeley, CA, USA) ECL Chemiluminescence Detection Kit, and the protein was quantified with ImageJ software.

### 2.8. Intestinal Microbial Analysis

After 15 weeks, the four experimental groups (*n* = 5) of fresh fecal samples were collected and stored at −80 °C for 16S rRNA bacterial genomic sequencing [25].

### 2.9. SCFAs in Feces Analysis

The analysis of SCFAs was performed by acidifying 100 mg of cecal contents with 20 μL of 50% dilute sulfuric acid (*v*/*v*), 995 uL of ether, and 5 uL of internal standard (1/100 μL of 2-ethylbutyric acid), followed by vortex mixing twice for 3 min and sonication in ice water for 30 min. Then, the mixture was stored at −20 °C for 30 min and centrifuged at 12,000× *g* for 15 min at 4 °C. The supernatant was vortexed with anhydrous sodium sulfate, recentrifuged, and filtered through a 0.22 µm membrane. The filtrate was subjected to analysis by gas chromatography coupled with GC (Shimadzu GC2010-plus, Kyoto, Japan), VF-WAXms column (30 m × 0.250 mm; 0.25 μm; Agilent Technologies, Signal, USA). Gas chromatography (GC) conditions were as follows: Injection volume of 1 μL; inlet temperature at 260 °C; distribution ratio of 50:1; and solvent delay for 2.5 min. The heating program was as follows: Initial temperature of 80 °C; program temperature of 120 °C at 40 °C/min; and temperature of 200 °C at 10 °C/min. After a hold time of 2 min and running time of 3 min, the mass spectrometry conditions were: Ion source temperature of 230 °C, quadrupole temperature of 150 °C, transfer line temperature of 250 °C, and electron energy of 70 eV. The scan mode was a full scan mode with a mass spectrum range of 30 to 300 *m*/*z*. Standard solutions included acetic acid, propionic acid, isobutyric acid, butyric acid, isovaleric acid, and caproic acid (Aladdin Biochemical Technology Co., Ltd., Shanghai, China). The quantitative analysis method involved comparing the peak area of the sample with the internal standard 2-ethylbutyric acid.

### 2.10. Statistical Analysis

Statistical analysis and Western blotting were performed using GraphPad Prism 7.00 software (La Jolla, CA, USA). All data are presented as mean ± SEM. Differences were determined to be statistically significant using the analysis of variance and Tukey’s multiple comparison test (*p* < 0.05 was considered statistically significant).

## 3. Results

### 3.1. Identification of Related Components in Crude Polysaccharides

The CIP was extracted from isinglass, and the yield was 31.33 mg/g. The total polysaccharide content of CIP was 32.12% (Figure S1). Gel permeation chromatography indicated that the average molecular weight of CIP is 3.97 × 10^3^ Da (Figure S2).

**Figure 1:**
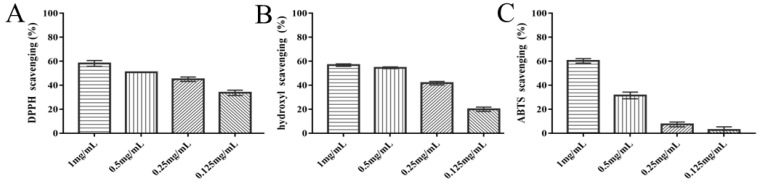
Antioxidant activity of CIP in vitro. (**A**) DPPH radical scavenging activity; (**B**) ABTS radical scavenging activity; (**C**) hydroxyl radical scavenging activity (*n* = 3).

Oxygen radicals can be scavenged directly or indirectly by polysaccharides. CIP provides a dose effect by removing DPPH, ABTS, and hydroxyl radicals. At 1 mg/mL, DPPH, ABTS, and hydroxyl radical removal rates reached 60%, respectively (Figure 1A–C). Therefore, the data revealed that CIP may scavenge free radicals in vitro and may possess antioxidant effects.

### 3.2. CIP Prevented Excessive Weight Gain in High-Fat Diet Mice

**Figure 2:**
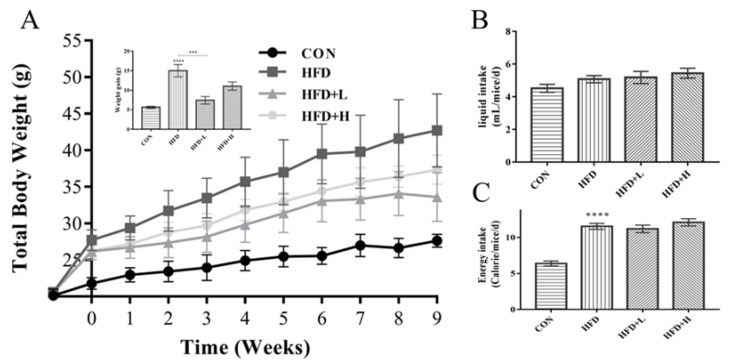
Changes in body weight gain, fluid intake, and energy intake of mice in each group. (**A**) Growth curve of total weight gain in each group (*n* = 7); (**B**) average fluid intake in each group (mL/mice/d); (**C**) average energy intake of each group (kcal/mice/d). Data were shown as mean ± SEM. *** *p* < 0.001, and **** *p* < 0.0001 compared with the other groups.

At the start of the experiment, the mice in each group were about the same weight. After 3 weeks, the average weight gain of the high-fat diet mice was clearly greater than the CON group mice, which indicates successful modeling (Figure 2A). At the end of 13 weeks, the HFD+L group had clearly lower body weight gain than the HFD group (*p* < 0.0001; Figure 2B). Body weight of the HFD+L group increased less than the HFD+H group. There were no significant differences in daily energy intake between groups except for the CON group (Figure 2B,C).

### 3.3. CIP Prevented Adipose Tissue Accumulation in High-Fat Diet Mice

**Figure 3:**
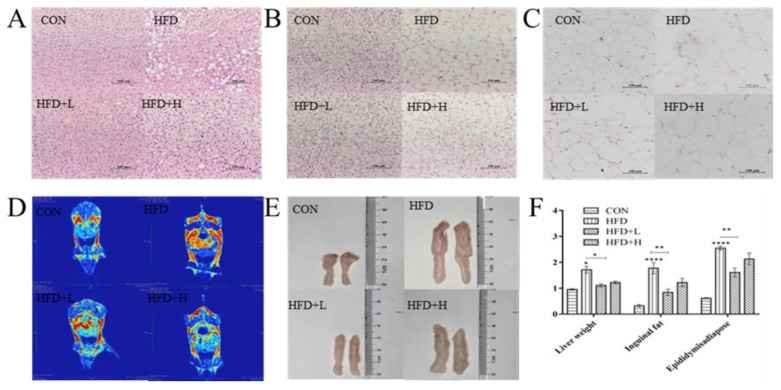
CIP prevented fat mass accumulation in HFD-fed mice. (**A**) H&E staining of liver, (**B**) H&E staining of brown fat and groin fat, and (**C**) of mice in CON, HFD, HFD+L, and HFD+H (scale bar, 100 μm); (**D**) quantitative magnetic resonance was performed for the analysis of body composition; (**E**) length of groin fat; (**F**) the organs weight of mice (*n* = 7). Data were shown as mean ± SEM. * *p* < 0.05, ** *p* < 0.005 and **** *p* < 0.0001 compared with the other groups.

Liver H&E-stained sections reveal that the number of white lipid droplets in the HFD+L group was clearly smaller than the HFD group, indicating the adverse accumulation of liver lipid (Figure 3A). The hepatic fat accumulation was clearly reduced after CIP intervention. At the end of 13 weeks, epididymis and groin fat weight increased clearly in the HFD group compared to the CON group (Figure 3E). In addition, we observed that CIP intervention reduced their weight, especially in the HFD+L group. The adipocyte size was clearly increased in the HFD group, and CIP intervention clearly reduced the increase in epididymis and cell size of brown fat induced by HFD (Figure 3B,C). Furthermore, the size and length of groin fat in HFD+L group were clearly reduced compared to the HFD group. Total body fat distribution was clearly reduced in the HFD+L group compared with the HFD group (Figure 3D). The inhibitory effect of HFD+H group on adipose tissue development was relatively less, which was consistent with the changing trend of body weight. These results suggest that CIP has a regulatory effect on body weight and adipose tissue in mice induced by the high-fat diet.

### 3.4. CIP Regulates Serum Hormones and Cytokines in High-Fat Diet Mice

**Figure 4:**
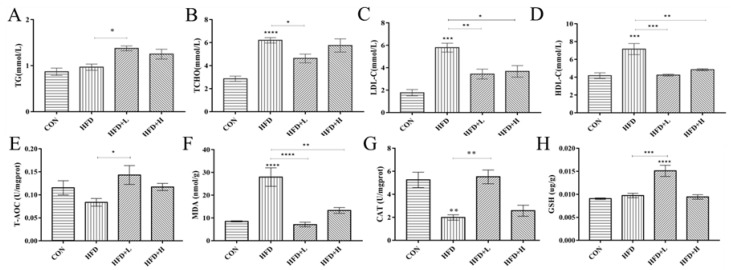
Effect of CIP on antioxidant activity in liver and serum lipid levels in HFD-fed mice (*n* = 5). (**A**) TG, (**B**) TCHO, (**C**) LDL-c, and (**D**) HDL-c in serum and (**E**) T-AOC, (**F**) MDA, (**G**) CAT, and (**H**) GSH in the liver of HFD-fed mice. Data were shown as mean ± SEM. * *p* < 0.05, ** *p* < 0.005, *** *p* < 0.001, and **** *p* < 0.0001 compared with the other groups.

**Figure 5:**
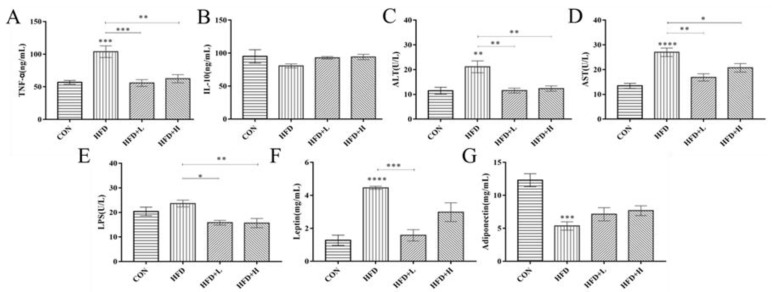
Effect of CIP on serum cytokine in HFD-fed mice (*n* = 5). (**A**) TNF-α, (**B**) IL-10, (**C**) ALT, (**D**) AST, (**E**) LPS, (**F**) leptin, and (**G**) adiponectin. Data were shown as mean ± SEM. * *p* < 0.05, ** *p* < 0.005, *** *p* < 0.001, and **** *p* < 0.0001 compared with the other groups.

Serum *TG* was increased in HFD+L group (Figure 4A,B) (*p* < 0.001) compared with the HFD group. Moreover, the *TCHO* level had a positive effect. Serum *LDL-C* concentration was clearly decreased in CIP group, especially in HFD+L group (Figure 4C) (*p* < 0.001). Similarly, CIP showed a reduction in *HDL-C* levels compared with the HFD group (Figure 4D). The changes in liver *GSH*, *T-AOC*, *CAT,* and *MDA* were detected after the intervention (Figure 4E–H). The HFD+L group showed higher concentrations of *T-AOC*, *CAT,* and *GSH* than the HFD group, whereas *MAD* had lower concentrations. Leptin, adiponectin, *LPS*, *AST,* and *ALT* were measured for changes in serum hormone levels. Compared with the CON group, leptin secreted by adipocytes in HFD group was clearly increased (*p* < 0.0001) (Figure 5F), while the *ADP* level was decreased (Figure 5G). Serum *LPS* level in CIP intervention group was lower than the HFD group (Figure 5E). This suggests that the high-fat diet induces leptin and lipopolysaccharide production and reduces *ADP* secretion in mice. In addition, serum *ALT* and *AST* levels were clearly increased in the HFD group, providing additional evidence for HFD-induced liver injury in mice. CIP intervention clearly reduced their serum levels, suggesting that CIP protects against liver damage induced by the HFD in mice. The level of *TNF-α* was clearly increased in HFD group (*p* < 0.001). The *TNF-α* level was clearly decreased after CIP administration. There was no statistical significance of *IL-10* in CIP-intervened mice (Figure 5B).

### 3.5. CIP Regulates the Expression Levels of Genes Involved in Lipid Metabolism and Inflammation in the Liver

**Figure 6:**
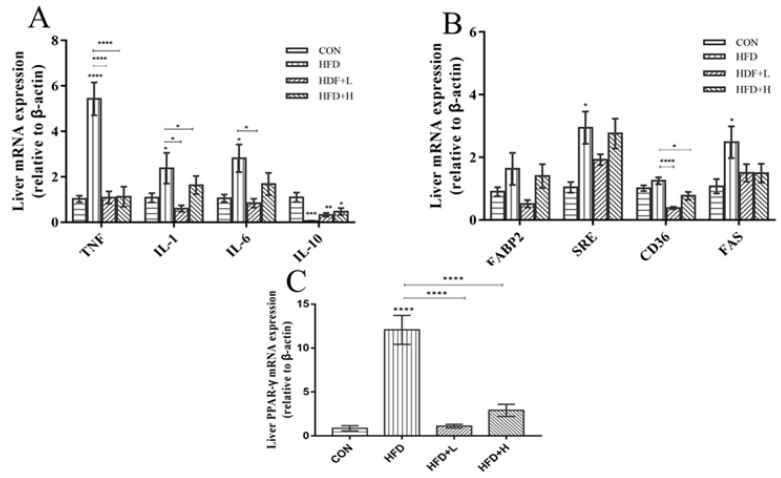
Effect of CIP on gene expression in liver (n = 5). (**A**) Lipid metabolism gene expression; (**B**) inflammatory gene expression; and (**C**) PPAR-γ. Data were shown as mean ± SEM. * *p* < 0.05, ** *p* < 0.005, *** *p* < 0.001, and **** *p* < 0.0001 compared with the other groups.

The experiment examined mRNA expression levels related to lipid metabolism and inflammation, including peroxisome proliferator-activated receptor γ (*PPAR-γ*), sterol regulatory element binding protein 1 (*Srebp1c*), platelet glycoprotein 4 (*CD36*), fatty acid binding protein (*Fabp2*), fatty acid synthase (*FAS*), *IL-1β*, *IL-6*, *IL-10,* and *TNF-α*, in order to investigate the molecular mechanism by which CIP inhibits hepatic lipid accumulation and inflammation. RT-qPCR results (Figure 6B) indicated that the expression levels of *FAS, Srebp1*, *CD36*, and *Fabp2* were increased in HFD mice, while the CIP treatment clearly improved these changes. In the HFD+L group, the expression level of *PPAR-γ* was clearly reduced and tended to the normal level (*p* < 0.0001) (Figure 6C), compared with the HFD group. In addition, the expression levels of *TNF-α, IL-1β,* and *IL-6* increased in HFD group (*p* < 0.005) (Figure 6A), while the expression level of anti-inflammatory factor *IL-10* decreased. The expression level of pro-inflammatory gene was clearly decreased, and the expression level of anti-inflammatory factor was upregulated after administration of CIP. The protective effect of CIP on lipid metabolism and inflammatory response-related gene expression was analyzed by total RNA in liver tissues.

### 3.6. CIP Regulates the Expression Levels of Related Proteins in Colon, Liver, and Adipocytes

**Figure 7:**
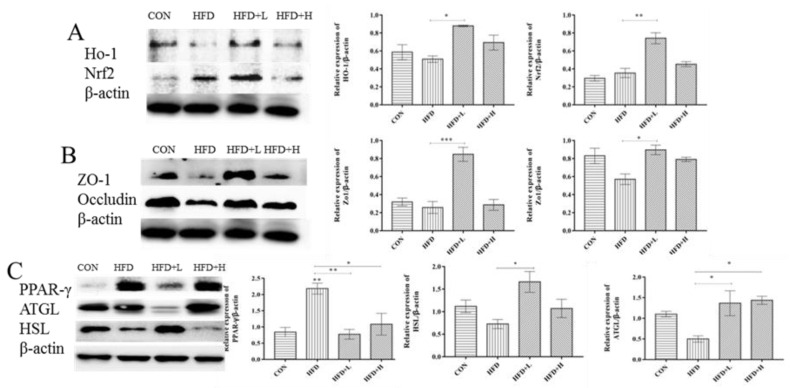
Effect of CIP on protein expression (*n* = 2~3). (**A**) Densitometric quantification and Western blot analysis of HO-1 and *Nrf2*; (**B**) densitometric quantification and Western blot analysis of *ZO-1* and Occludin; (**C**) densitometric quantification and Western blot analysis of PPAR-γ, ATGL, and HSL. Data were shown as mean ± SEM. * *p* < 0.05, ** *p* < 0.005 and *** *p* < 0.001 compared with the other groups.

In the experiment, Western blot was used to explore the expression levels of lipid metabolism proteins, including *PPAR-γ*, *ATGL*, *HSL*, liver antioxidant *Nrf2*, *HO-1*, intestinal tight junction proteins *ZO-1,* and *Occludin*. The data revealed that the expression level of *HSL* protein in HFD+L group was higher than the HFD group (*p* < 0.05) (Figure 7C), and the expression level of *ATGL* protein was clearly lower than the HFD group (*p* < 0.05) (Figure 7C). Compared with the HFD group, CIP intervention clearly reduced the expression level of *PPAR-γ*, among which the HFD+L group (*p* < 0.005) (Figure 7C) showed the clearest difference. CIP intervention had a protective effect on the intestinal tract of mice, and in the HFD+L group, the expression level of *ZO-1* was significantly clearly higher than the HFD group (*p* < 0.001) (Figure 7B). The expression level of *Occludin* in HFD+L group was clearly higher than the HFD group (*p* < 0.05) (Figure 7B). CIP intervention enhances the expression levels of *HO-1* and *Nrf2* in mice liver (Figure 7A).

### 3.7. CIP Improved the Diversity and Balance of Intestinal Flora in High-Fat Diet Mice

**Figure 8:**
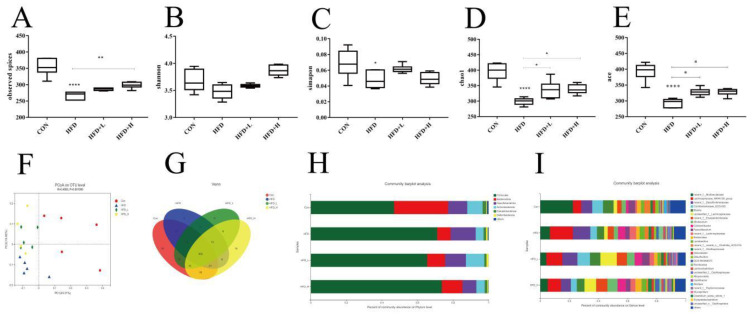
CIP increases the diversity and balance of (**A**–**E**) and α-diversity: The diversity parameters of observed species, Shannon’s diversity parameter, Simpson’s diversity parameter, Chao1’s diversity parameter, and ACE’s diversity parameter; (**F**) PCoA diagram based on weighted infraction; (**G**) OTUs of Venn diagram between processes; (**H**) relative abundance of intestinal microbiota at phylum level; (**I**) relative abundance of intestinal microbiota at genus level. Data (*n* = 5) were shown as mean ± SEM. * *p* < 0.05, ** *p* < 0.005, and **** *p* < 0.0001 compared with the other groups.

The values of species observed in the HFD group were clearly lower (*p* < 0.0001) (Figure 8A), compared with the CON group. In addition, the ACE index in the HFD group showed a significant downward trend (*p* < 0.0001) (Figure 8E). The data indicate that the intestinal flora richness of HFD mice decreased. In contrast, CIP intervention increased the diversity of the mice microbiota. Compared with HFD, the α-diversity (Chao1 and ACE diversity parameters) of mice in HFD+L group were clearly upregulated (*p* < 0.005) (Figure 8D,E). Although there were some changes in *Shannon* and *Simpson* indices after CIP intervention, there was no significant difference (Figure 8B,C). Only the HFD group showed clearly higher Simpson index than the HFD group. To better compare the structural changes in mice microbes between the four groups, PCoA was performed on the OTU abundances obtained from the four groups of samples. The PCoA diagram shows the similarity of mice microbes between the CON and HFD+L groups, while the data of the HFD and HFD+L groups were dislocated and located in two different regions (Figure 8F). In general, CIP intervention mitigated HFD-induced intestinal microbiota interference in mice.

**Figure 9:**
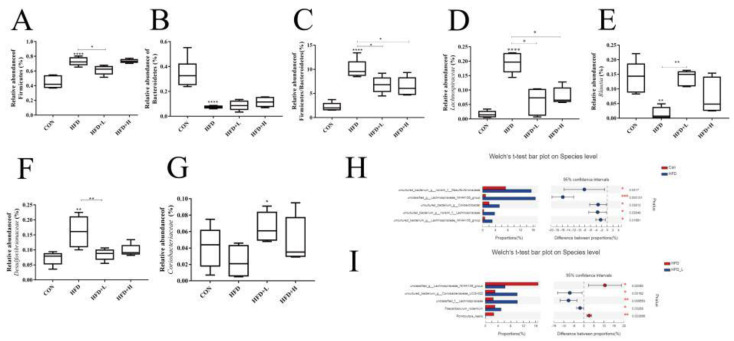
CIP improved the composition of intestinal flora in HFD-fed mice. (**A**) Relative abundance of Firmicutes; (**B**) relative abundance of Bacteroidetes; (**C**) relative abundance ratio of Firmicutes to Bacteroidetes; (**D**–**G**) abundance of intestinal bacteria in mice ((**D**), *Lachnospiraceae;* (**E**), *Blautia*; (**F**), *Desulfovibrionaceae*; (**G**), *Coriobacteriaceae*); (**H**) species-level correlation of microbiota between the HFD group and CON group; (**I**) species-level correlation of microbiota between the HFD group and HFD+L group. Data (*n* = 5) were shown as mean ± SEM. * *p* < 0.05, ** *p* < 0.005, and **** *p* < 0.0001 compared with the other groups.

At the level of phyla, *Firmicutes*, *Bacteroidetes, Deironyobacteria*, *Proteobacteria*, and *Actinomycetes* are mainly involved (Figure 8H). Compared with the HFD group, the relative abundance of *Firmicutes* in the intestine of mice in HFD group was clearly increased, but *Bacteroidetes* were clearly decreased (*p* < 0.0001) (Figure 9A,B). However, the *Firmicutes* decreased and *Bacteroidetes* increased in CIP-intervened mice compared with HFD, and the *Firmicutes/Bacteroidetes* ratio showed a similar pattern of change (Figure 9C).

At the genus level, the effects of CIP on intestinal microorganisms of HFD mice were further examined. CIP intervention in the HFD+L group reduced the relative abundance of *Lachnospiraceae_NK4A136* compared with the HFD group (*p* < 0.05) (Figure 9D). The relative abundance of anti-inflammatory bacteria *Blautia* increased clearly after CIP intervention (*p* < 0.005) (Figure 9E). In addition, the relative abundance of *Desulfovibrionaceae* was studied (*p* < 0.005) (Figure 9F). Compared with the HFD group, CIP intervention clearly reduced its relative abundance. Moreover, CIP intervention clearly increased the relative abundance of *Coriobacteriaceae* in HFD group (*p* < 0.05) (Figure 9G).

At the species level, the relative abundance of the CON and HFD groups was compared and the top five marker species of HFD group were *Bacterium-Desulfovibrionaceae*, *Lachnospiraceae_NK4A136*, *Bacterium-Lachnospiraceae*, *Bacterium-Colidextribacter*, and Bacterium-*Lachnospiraceae* (Figure 9H). However, by comparison between the HFD group and HFD+L group, the marked species of HFD+L group were *Bacterium-Coriobacteriaceae-UCG-002*, *Lachnospiraceae,* and *Faecalibaculum_rodentium*. The marked species in the model group were *Lachnospiraceae_NK4A136* and *Romboutsia-ilealis* (Figure 9I).

### 3.8. CIP Improved SCFAs Content in Cecum of High-Fat Diet Mice

**Figure 10:**
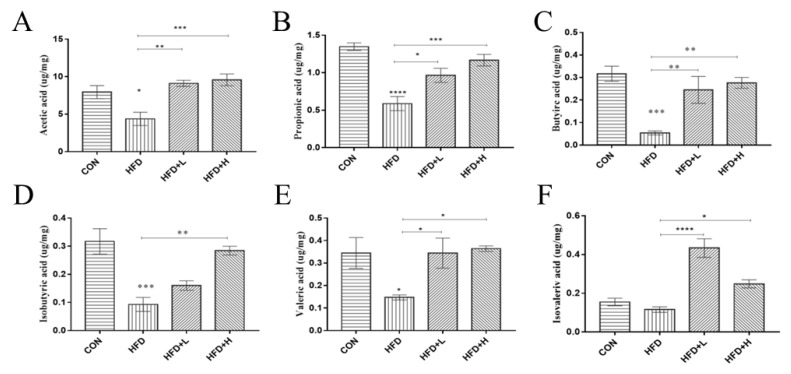
Colon content of SCFAs in each group after CIP intervention. (**A**) acetate, (**B**) propionate, (**C**) butyrie, (**D**) isobutyrie, (**E**) valerate and (**F**) isovalerate in the colon contents. Data (*n* = 5) were shown as mean ± SEM. * *p* < 0.05, ** *p* < 0.05, *** *p* < 0.001, and **** *p* < 0.0001 compared with the other groups.

SCFAs, including acetic acid, propionic acid, isobutyric acid, butyric acid, isovaleric acid, and valeric acid, are reported to have multiple beneficial effects during metabolism and are essential for protecting the intestinal barrier. Therefore, SCFAs levels of HFD mice treated with CIP were analyzed. The levels of acetate and butyrate in HFD+L group were clearly increased (*p* < 0.05) (Figure 10A,C), compared with the HFD group. Propionic acid and valerate acid levels were clearly increased after CIP intervention (*p* < 0.05) (Figure 10B,E). Similarly, isobutyric acid and isovaleric acid levels were clearly reduced in HFD mice, but were clearly reversed after CIP intervention (Figure 10D,F).

## 4. Discussion

Polysaccharides have been shown to be effective in the intervention of obesity [26]. Their anti-obesity mechanisms typically involve appetite suppression, reduced lipid absorption, promotion of lipolysis, reduced lipogenesis, or modulation of gut microbiota [13]. In this experiment, the whole-body adipose tissue of HFD+L mice was clearly reduced, especially the inguinal fat weight and cell size of HFD+L mice (Figure 3E,F). CIP intervention clearly ameliorated HFD-induced liver damage, such as hepatocyte fat accumulation and hepatocyte balloon degeneration, and restored a similar state to normal mice. This suggests that CIP intervention helps in improving obesity by reducing fat accumulation in the liver and adipose tissue rather than reducing appetite.

The HFD group had clearly increased serum *TCHO* and *LDL-C* levels as well as decreased *TG* levels throughout the experimental period, which was consistent with previous reports [8]. The levels of *LDL-C* and *TCHO* in serum of mice treated with CIP were lower (Figure 4B,C) compared with the HFD group. It may be that CIP intervention increases cholesterol excretion or reduces cholesterol synthesis, improving lipid metabolism disorders. In contrast, *TG* levels in the intervention group decreased since lipid parameters could not be normalized. In this study, the levels of *HDL-C* in the HFD group were clearly higher than those in the treatment and standard diet groups, which is consistent with a recent report [27,28]. The role of *HDL-C* has been challenged by the negative results of multiple studies on *HDL-C*-elevating drugs, raising paradoxes about the value of *HDL-C* as a risk biomarker and therapeutic target [29]. The results of this experiment are more likely to match the situation of HDL-C studies. Obesity is often associated with systemic inflammation, and oxidative stress is an important factor in colon inflammation [30]. The overproduction of *MDA* and the decrease in antioxidant activity of *GSH*, *CAT*, and *T-AOC* are the main characteristics of oxidative stress [31]. Lower doses of CIP inhibited these declines in liver antioxidant function indices more clearly than mice with higher doses (Figure 4E–H) (*p* < 0.05). It may be related to achieving the right dose of CIP to have a strong antioxidant effect.

Leptin is a specialized secreted protein in adipose tissue that regulates food intake and energy expenditure [32]. The study found that leptin levels increased in mice in response to pro-inflammatory stimuli [33], such as *TNF* and *LPS* [34]. Therefore, in the HFD group, it is speculated that the elevated leptin level was mainly due to the increased secretion/expression levels of *TNF-α* and *LPS*. CIP intervention clearly improved this phenomenon. Adiponectin, a small amount of anti-inflammatory factor secreted by adipose tissue, stimulates autophagy and reduces oxidative stress. In the current study, we found that high-fat diet mice had decreased serum adiponectin levels and increased *TNF-α* and *LPS*, and CIP intervention clearly ameliorated these changes. It can be seen that the degree of inflammatory response is closely related to the secretion of leptin and adiponectin. Furthermore, CIP intervention clearly prevented these alterations in the liver. In addition, in high-fat diet mice, serum *AST* and *ALT* (Figure 5C,D) levels showed an increasing trend, which was clearly downregulated to the levels of normal mice by the CIP treatment.

At the same time, the expression level of host genes closely related to lipid metabolism was studied. In this study, it was found that the mRNA expression levels of *PPAR-γ*, *CD36*, *Fabp2*, *Srebp-1c*, and *FAS* were extremely expressed in the liver of high-fat diet mice. CIP intervention reversed the upregulation of these genes in obese mice (Figure 6B), indicating that CIP intervention reduced lipid synthesis, which also corresponds to restoration of fatty liver morphology and inguinal fat expansion, compared with the HFD group. The inflammatory program, activated at the onset of adipose tissue expansion and subsequently in chronic obesity, continually shifts the immune system to a pro-inflammatory phenotype [35]. The results showed that *TNF-α*, *IL-1β,* and *IL-6* levels were clearly increased in HFD group, while *IL-10* levels were decreased (Figure 6A). This suggests that the high-fat diet in mice induces low systemic inflammation. In this case, CIP supplementation clearly reduced inflammation. Therefore, the potential mechanism by which CIP reduces obesity may be through reducing inflammation and lipid synthesis.

Studies have shown that activation of the *Nrf2/HO-1* signaling pathway may promote the expression of *ZO-1* protein in intestinal epithelial cells [36]. Furthermore, *Nrf2* helped in restoring the levels of *Occludin* and *ZO-1* in inflammatory mice [37]. Therefore, we investigated the expression level of liver-related proteins to investigate whether CIP could increase the expression level of tight junction proteins in mice through the Nrf2/HO-1 signaling pathway. Compared with the model group, the relative expression levels of *HO-1* and *Occludin* in the HFD+L group were clearly increased (*p* < 0.05) (Figure 7B). Meanwhile, the CIP treatment also promoted *Nrf2/HO-1* signal transduction (Figure 7A). Recent studies have shown that the *Nrf2/HO-1* signaling pathway regulates a variety of antioxidant and inflammatory mechanisms. Furthermore, *ATGL/HSL*-mediated lipid degradation directly or indirectly affects PPAR-γ signaling through the master transcriptional regulators of adipogenesis, resulting in decreased lipid synthesis and storage. This is shown by a growing body of research in [38]. Compared with the HFD group, high-fat diet mice treated with CIP showed reduced *AGTL* expression in groin fat (Figure 7C). At the same time, *PPAR-γ* expression was decreased in groin fat of high-fat mice treated with CIP, suggesting that CIP-mediated lipolysis was associated with inhibition of *PPAR-γ* target gene expression. In conclusion, our results suggest that CIP reverses high-fat diet-induced obesity by reducing *ATGL*-mediated lipolysis of fat cells.

Obesity induced by fat diet is closely related to intestinal microbiome disorders. In the HFD group, Chao1 and ACE indices decreased clearly (Figure 8D,E), while Shannon and Simpson indices increased (Figure 8B,C). CIP intervention in HFD-fed mice counteracted the interference with richness and diversity. PCoA analysis showed that the bacterial community in HFD and HFD+L groups was distant (Figure 8F), indicating that the HFD+L group alleviated the bacterial community disorder in HFD-fed mice. These observations suggest that CIP intervention mitigated the reduction in intestinal flora richness and diversity induced by the high-fat diet. Particularly in the HFD+L group, this may eventually lead to improvements in obesity-related characteristics. The balance between *Firmicutes* and *Bacteroidetes* may play an important role in obesity-related inflammation [39]. Recent studies have shown that the number of *Bacteroidetes* in obese mice is lower, while the number of *Firmicutes* is higher. Human experiments on obese and healthy subjects also show the same trend of *Bacteroides* as animal experiments [17]. The frequency of *Bacteroides* was clearly lower in the HFD+L group compared with the HFD group (*p* < 0.0001) (Figure 9B). However, CIP intervention alleviated this condition, but not statistically. The proportion of *Firmicutes* in the HFD+L group was 20% lower than the HFD group (Figure 9A). Furthermore, the F/B ratio increased proportionally in the HFD group compared with the CON group (Figure 9C). These results suggest that CIP intervention can counteract the increased *Firmicutes* level and F/B ratio in HFD-fed mice and relieve intestinal disorders.

A recent study showed that seven families including *Lachnospiraceae* and *Desulfovibrionaceae* were enriched in HFD-fed mice [33]. In addition, members of *Lachnospiraceae* induced an increase in liver and adipose tissue weight in germ-free mice, indicating that the family plays an important role in obesity [40]. *Desulfovibrionaceae* produces endotoxins and participates in the pathogenesis of intestinal inflammatory diseases [41]. However, *Blautia* may help reduce inflammation associated with obesity-related complications. This suggests that CIP reduces HFD-induced chronic inflammation and fat accumulation, accompanied by an increase in beneficial bacteria (*Blautia*) and a decrease in pathogenic bacteria (*Desulphurvibrio* and *Lachnospiraceae*).

SCFAs, as metabolites of intestinal microorganisms, have a significant impact on peripheral tissues. Previous research has highlighted the importance of SCFAs in improving inflammatory disease and promoting colon cell health [42]. In this study, CIP intervention produced more short-chain fatty acids in obese mice. *Bacteroides*, as members of the polysaccharide degradation consortium, are likely to be the main source of propionate. Among them, propionate can prevent weight gain and lipid content in liver cells. SCFAs metabolism is one of the potential mechanisms of polysaccharide treatment to reduce inflammation in mice [43]. The results support that CIP ameliorates obesity in mice by correcting intestinal microbial metabolic disorders induced by HFD.

To evaluate the efficacy of CIP against obesity, different doses (25 and 50 mg/kg) were administered. At 50 mg/kg, an increase in the release of inflammatory cytokines was observed, which affected intestinal tight junction protein expression. Therefore, we can consider a dose of 25 mg/kg to be optimal, which is similar to previous studies [44].

In this study, we demonstrated that CIP works only to prevent the development of obesity, when the individual receives a high-fat diet. Further work is required to demonstrate the therapeutic effects of CIP, which is to reduce body weight in an individual who is already overweight.

## 5. Conclusions

In conclusion, this study demonstrated that the CIP treatment decreased the levels of TC, HDL-C, and LDL-C and increased the levels of TG in mice, suggesting that the CIP intervention increased cholesterol excretion or reduced cholesterol synthesis. CIP had antioxidant effects in DPPH, hydroxyl radical, and ABTS experiments, and the levels of T-AOC, GSH, and CAT in liver of mice were clearly increased, while the content of MDA was clearly decreased. Meanwhile, the results of HO-1/Nrf2 signaling pathway showed that CIP had antioxidant effects, indicating that CIP improved the ability of anti-oxidative stress in mice. Pathological sections showed a decrease in the size of white lipid droplets and fat cells in liver cells of mice treated with CIP. Obesity is associated with systemic, chronic, and mild inflammation. The results showed that CIP decreased the serum levels of inflammatory cytokine TNF-α and the mRNA expression levels of IL-6, IL-1β, and TNF-α in mice, suggesting that CIP improved inflammation during the development of obesity. CIP reversed the upregulation of genes in obese mice, suggesting that CIP reduced lipid synthesis or metabolism. The results showed that CIP clearly affected the expression level of intestinal tight junction protein in mice. The results of intestinal microbiota showed that CIP decreased *Firmicutes* abundance and increased *Bacteroidetes* abundance. In particular, CIP intervention increased the abundance of *Blautia* and *Coriobacteriaceae*. Compared with the HFD group, CIP increased the intestinal short-chain fatty acid content of mice, which also indicated that it alleviated the occurrence of inflammation. Therefore, CIP may exhibit anti-obesity effects through its ability to modulate the mice microbiota, reduce inflammation, and protect the gut barrier.

## Supplementary Materials

The following supporting information can be downloaded at: https://www.mdpi.com/article/10.3390/nu14193928/s1, Figure S1: Standard curve of glucose content; Table S1: Molecular weight standard curve.

## References

[1] Moayyedi P. (2008). The epidemiology of obesity and gastrointestinal and other diseases: An overview. Dig. Dis. Sci..

[2] Zhang Y., Zhao N., Yang L., Hong Z., Cai B., Le Q., Yang T., Shi L., He J., Cui C. (2021). Insoluble dietary fiber derived from brown seaweed Laminaria japonica ameliorate obesity-related features via modulating gut microbiota dysbiosis in high-fat diet-fed mice. Food Funct..

[3] Lu J., Zhu M., Zhang H., Liu H., Xia B., Wang Y., Shi X., Peng L., Wu J. (2020). Neohesperidin attenuates obesity by altering the composition of the gut microbiota in high-fat diet-fed mice. FASEB J. Off. Publ. Fed. Am. Soc. Exp. Biol..

[4] Rossi F., Bellini G., Luongo L., Manzo I., Tolone S., Tortora C., Bernardo M., Grandone A., Conforti A., Docimo L. (2016). Cannabinoid Receptor 2 as Antiobesity Target: Inflammation, Fat Storage, and Browning Modulation. J. Clin. Endocrinol. Metab..

[5] Chang M., Yang Z., Yang S. (2020). Roles of Adipokines in Digestive Diseases: Markers of Inflammation, Metabolic Alteration and Disease Progression. Int. J. Mol. Sci..

[6] Jung U., Choi M. (2014). Obesity and its metabolic complications: The role of adipokines and the relationship between obesity, inflammation, insulin resistance, dyslipidemia and nonalcoholic fatty liver disease. Int. J. Mol. Sci..

[7] Ohashi K., Shibata R., Murohara T., Ouchi N. (2014). Role of anti-inflammatory adipokines in obesity-related diseases. Trends Endocrinol. Metab. TEM.

[8] Zhong H., Abdullah, Deng L., Zhao M., Tang J., Liu T., Zhang H., Feng F. (2020). Probiotic-fermented blueberry juice prevents obesity and hyperglycemia in high fat diet-fed mice in association with modulating the gut microbiota. Food Funct..

[9] Schoultz I., Keita Å. (2019). Cellular and Molecular Therapeutic Targets in Inflammatory Bowel Disease-Focusing on Intestinal Barrier Function. Cells.

[10] Piotrowska M., Swierczynski M., Fichna J., Piechota-Polanczyk A. (2021). The *Nrf2* in the pathophysiology of the intestine: Molecular mechanisms and therapeutic implications for inflammatory bowel diseases. Pharmacol. Res..

[11] Ringseis R., Gessner D., Eder K. (2020). The Gut-Liver Axis in the Control of Energy Metabolism and Food Intake in Animals. Annu. Rev. Anim. Biosci..

[12] Koh A., De Vadder F., Kovatcheva-Datchary P., Bäckhed F. (2016). From Dietary Fiber to Host Physiology: Short-Chain Fatty Acids as Key Bacterial Metabolites. Cell.

[13] Cuevas-Sierra A., Ramos-Lopez O., Riezu-Boj J., Milagro F., Martinez J. (2019). Diet, Gut Microbiota, and Obesity: Links with Host Genetics and Epigenetics and Potential Applications. Adv. Nutr..

[14] De Vadder F., Kovatcheva-Datchary P., Goncalves D., Vinera J., Zitoun C., Duchampt A., Bäckhed F., Mithieux G. (2014). Microbiota-generated metabolites promote metabolic benefits via gut-brain neural circuits. Cell.

[15] Steliou K., Boosalis M., Perrine S., Sangerman J., Faller D. (2012). Butyrate histone deacetylase inhibitors. BioResearch Open Access.

[16] Frost G., Sleeth M., Sahuri-Arisoylu M., Lizarbe B., Cerdan S., Brody L., Anastasovska J., Ghourab S., Hankir M., Zhang S. (2014). The short-chain fatty acid acetate reduces appetite via a central homeostatic mechanism. Nat. Commun..

[17] Turnbaugh P., Ley R., Mahowald M., Magrini V., Mardis E., Gordon J. (2006). An obesity-associated gut microbiome with increased capacity for energy harvest. Nature.

[18] Maruvada P., Leone V., Kaplan L., Chang E. (2017). The Human Microbiome and Obesity: Moving beyond Associations. Cell Host Microbe.

[19] Clemente J., Ursell L., Parfrey L., Knight R. (2012). The impact of the gut microbiota on human health: An integrative view. Cell.

[20] Anhê F., Nachbar R., Varin T., Trottier J., Dudonné S., Le Barz M., Feutry P., Pilon G., Barbier O., Desjardins Y. (2019). Myrciaria dubiaTreatment with camu camu (*Myrciaria dubia*) prevents obesity by altering the gut microbiota and increasing energy expenditure in diet-induced obese mice. Gut.

[21] Koboziev I., Scoggin S., Gong X., Mirzaei P., Zabet-Moghaddam M., Yosofvand M., Moussa H., Jones-Hall Y., Moustaid-Moussa N. (2020). Effects of Curcumin in a Mouse Model of Very High Fat Diet-Induced Obesity. Biomolecules.

[22] Xie J., Jin M., Morris G., Zha X., Chen H., Yi Y., Li J., Wang Z., Gao J., Nie S. (2016). Advances on Bioactive Polysaccharides from Medicinal Plants. Crit. Rev. Food Sci. Nutr..

[23] Chen Y., Liu Y., Sarker M., Yan X., Yang C., Zhao L., Lv X., Liu B., Zhao C. (2018). Structural characterization and antidiabetic potential of a novel heteropolysaccharide from Grifola frondosa via IRS1/PI3K-JNK signaling pathways. Carbohydr. Polym..

[24] Liu C., Cui Y., Pi F., Guo Y., Cheng Y., Qian H. (2019). Torularhodin Ameliorates Oxidative Activity in Vitro and d-Galactose-Induced Liver Injury via the Nrf2/HO-1 Signaling Pathway in Vivo. J. Agric. Food Chem..

[25] Wan Y., Wang F., Yuan J., Li J., Jiang D., Zhang J., Li H., Wang R., Tang J., Huang T. (2019). Effects of dietary fat on gut microbiota and faecal metabolites, and their relationship with cardiometabolic risk factors: A 6-month randomised controlled-feeding trial. Gut.

[26] Li J., Pang B., Shao D., Jiang C., Hu X., Shi J. (2020). Artemisia sphaerocephala Krasch polysaccharide mediates lipid metabolism and metabolic endotoxaemia in associated with the modulation of gut microbiota in diet-induced obese mice. Int. J. Biol. Macromol..

[27] Pothuraju R., Sharma R., Kavadi P., Chagalamarri J., Jangra S., Bhakri G., De S. (2016). Anti-obesity effect of milk fermented by Lactobacillus plantarum NCDC 625 alone and in combination with herbs on high fat diet fed C57BL/6J mice. Benef. Microbes.

[28] Lee E., Jung S., Lee S., Lee N., Paik H., Lim S. (2018). Lactobacillus plantarum Strain Ln4 Attenuates Diet-Induced Obesity, Insulin Resistance, and Changes in Hepatic mRNA Levels Associated with Glucose and Lipid Metabolism. Nutrients.

[29] Xiang A., Kingwell B. (2019). Rethinking good cholesterol: A clinicians’ guide to understanding HDL. Lancet Diabetes Endocrinol..

[30] Di Sabatino A., Santilli F., Guerci M., Simeone P., Ardizzone S., Massari A., Giuffrida P., Tripaldi R., Malara A., Liani R. (2016). Oxidative stress and thromboxane-dependent platelet activation in inflammatory bowel disease: Effects of anti-TNF-α treatment. Thromb. Haemost..

[31] Li H., Shen L., Lv T., Wang R., Zhang N., Peng H., Diao W. (2019). Salidroside attenuates dextran sulfate sodium-induced colitis in mice via SIRT1/FoxOs signaling pathway. Eur. J. Pharmacol..

[32] Pan W., Myers M. (2018). Leptin and the maintenance of elevated body weight. Nat. Rev. Neurosci..

[33] Liu B., Zhang Y., Wang R., An Y., Gao W., Bai L., Li Y., Zhao S., Fan J., Liu E. (2018). Western diet feeding influences gut microbiota profiles in apoE knockout mice. Lipids Health Dis..

[34] Grunfeld C., Zhao C., Fuller J., Pollack A., Moser A., Friedman J., Feingold K. (1996). Endotoxin and cytokines induce expression of leptin, the ob gene product, in hamsters. J. Clin. Investig..

[35] Saltiel A., Olefsky J. (2017). Inflammatory mechanisms linking obesity and metabolic disease. J. Clin. Investig..

[36] Liu Y., Bao Z., Xu X., Chao H., Lin C., Li Z., Liu Y., Wang X., You Y., Liu N. (2017). Extracellular Signal-Regulated Kinase/Nuclear Factor-Erythroid2-like2/Heme Oxygenase-1 Pathway-Mediated Mitophagy Alleviates Traumatic Brain Injury-Induced Intestinal Mucosa Damage and Epithelial Barrier Dysfunction. J. Neurotrauma.

[37] Lau W., Liu S., Pahlevan S., Yuan J., Khazaeli M., Ni Z., Chan J., Vaziri N. (2015). Role of *Nrf2* dysfunction in uremia-associated intestinal inflammation and epithelial barrier disruption. Dig. Dis. Sci..

[38] Schreiber R., Hofer P., Taschler U., Voshol P., Rechberger G., Kotzbeck P., Jaeger D., Preiss-Landl K., Lord C., Brown J. (2015). Hypophagia and metabolic adaptations in mice with defective ATGL-mediated lipolysis cause resistance to HFD-induced obesity. Proc. Natl. Acad. Sci. USA.

[39] Rosenbaum M., Knight R., Leibel R. (2015). The gut microbiota in human energy homeostasis and obesity. Trends Endocrinol. Metab. TEM.

[40] Kameyama K., Itoh K. (2014). Intestinal colonization by a Lachnospiraceae bacterium contributes to the development of diabetes in obese mice. Microbes Environ..

[41] Jiang P., Zheng W., Sun X., Jiang G., Wu S., Xu Y., Song S., Ai C. (2021). Sulfated polysaccharides from Undaria pinnatifida improved high fat diet-induced metabolic syndrome, gut microbiota dysbiosis and inflammation in BALB/c mice. Int. J. Biol. Macromol..

[42] Chapman M., Grahn M., Giamundo P., O’Connell P., Onwu D., Hutton M., Maudsley J., Norton B., Rogers J., Williams N. (1993). New technique to measure mucosal metabolism and its use to map substrate utilization in the healthy human large bowel. Br. J. Surg..

[43] Liu C., Hua H., Zhu H., Cheng Y., Guo Y., Yao W., Qian H. (2021). Aloe polysaccharides ameliorate acute colitis in mice via Nrf2/HO-1 signaling pathway and short-chain fatty acids metabolism. Int. J. Biol. Macromol..

[44] Kerboua K.A., Benosmane L., Namoune S., Ouled-Diaf K., Ghaliaoui N., Bendjeddou D. (2021). Anti-inflammatory and antioxidant activity of the hot water-soluble polysaccharides from *Anacyclus pyrethrum* (L.) Lag. roots. J. Ethnopharmacol..

