# Supplementary figures and images for: Isinglass Polysaccharides Regulate Intestinal-Barrier Function and Alleviate Obesity in High-Fat Diet Mice through the HO-1/Nrf2 Pathway and Intestinal Microbiome Environment
Source: Nutrients. 2022 Sep 22;14(19):3928. doi: 10.3390/nu14193928 (PMC9573006; doi:10.3390/nu14193928)

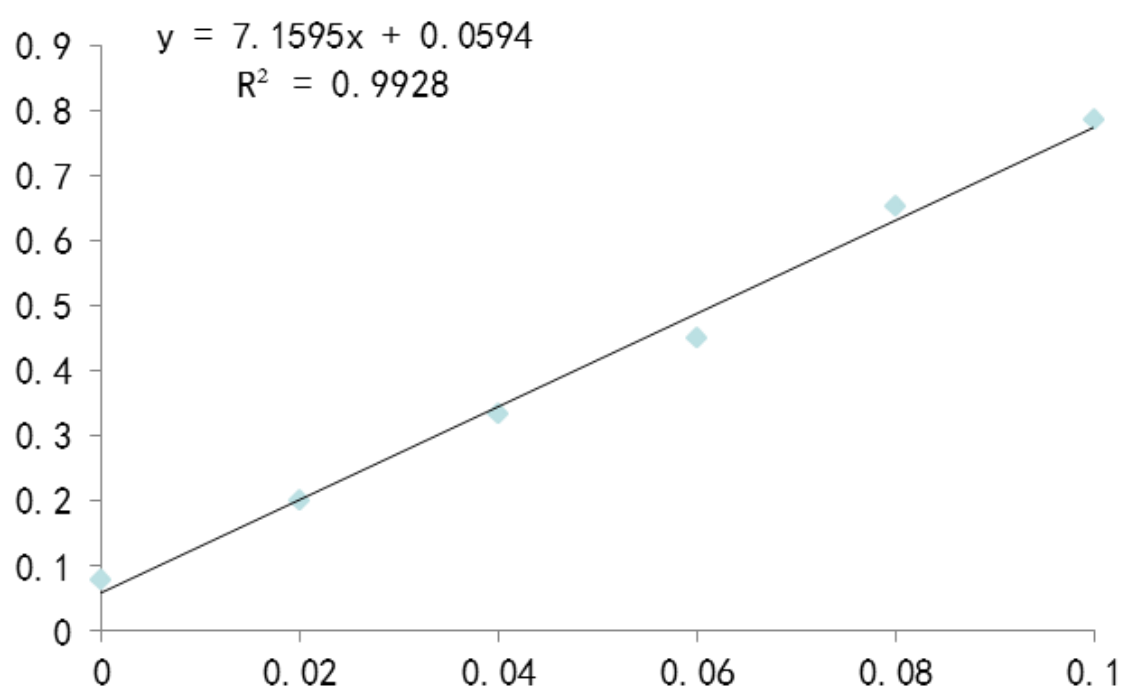

**Figure S1.** Standard curve of glucose content

Supplement: Supplementary file 1 [file nutrients-14-03928-s001.zip › supplementary Figure S1.pdf]

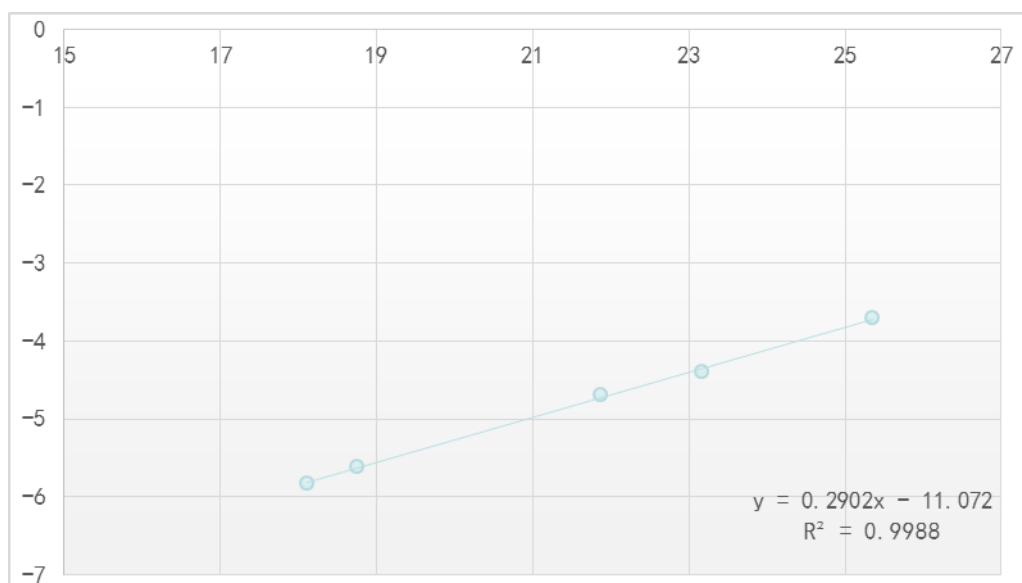

**Figure S2.** Molecular weight standard curve.

Supplement: Supplementary file 1 [file nutrients-14-03928-s001.zip › supplementary Figure S2.pdf]
